# Alterations of Sleep and Sleep Oscillations in the Hemiparkinsonian Rat

**DOI:** 10.3389/fnins.2019.00148

**Published:** 2019-02-25

**Authors:** Jelena Ciric, Slobodan Kapor, Milka Perovic, Jasna Saponjic

**Affiliations:** ^1^Department of Neurobiology, Institute for Biological Research “Siniša Stanković”, University of Belgrade, Belgrade, Serbia; ^2^School of Medicine, University of Belgrade, Belgrade, Serbia

**Keywords:** sleep, electroencephalography, sleep spindle, high voltage sleep spindle, substantia nigra pars compacta, oscillations, motor cortex, hippocampus

## Abstract

Our previous studies in the rat model of Parkinson’s disease (PD) cholinopathy demonstrated the sleep-related alterations in electroencephalographic (EEG) oscillations at the cortical and hippocampal levels, cortical drives, and sleep spindles (SSs) as the earliest functional biomarkers preceding hypokinesia. Our aim in this study was to follow the impact of a unilateral substantia nigra pars compacta (SNpc) lesion in rat on the cortical and hippocampal sleep architectures and their EEG microstructures, as well as the cortico-hippocampal synchronizations of EEG oscillations, and the SS and high voltage sleep spindle (HVS) dynamics during NREM and REM sleep. We performed unilateral SNpc lesions using two different concentrations/volumes of 6-hydroxydopamine (6-OHDA; 12 μg/1 μl or 12 μg/2 μl). Whereas the unilateral dopaminergic neuronal loss >50% throughout the overall SNpc rostro-caudal dimension prolonged the Wake state, with no change in the NREM or REM duration, there was a long-lasting theta amplitude augmentation across all sleep states in the motor cortex (MCx), but also in the CA1 hippocampus (Hipp) during both Wake and REM sleep. We demonstrate that SS are the hallmarks of NREM sleep, but that they also occur during REM sleep in the MCx and Hipp of the control rats. Whereas SS are always longer in REM vs. NREM sleep in both structures, they are consistently slower in the Hipp. The dopaminergic neuronal loss increased the density of SS in both structures and shortened them in the MCx during NREM sleep, without changing the intrinsic frequency. Conversely, HVS are the hallmarks of REM sleep in the control rats, slower in the Hipp vs. MCx, and the dopaminergic neuronal loss increased their density in the MCx, but shortened them more consistently in the Hipp during REM sleep. In addition, there was an altered synchronization of the EEG oscillations between the MCx and Hipp in different sleep states, particularly the theta and sigma coherences during REM sleep. We provide novel evidence for the importance of the SNpc dopaminergic innervation in sleep regulation, theta rhythm generation, and SS/HVS dynamics control. We suggest the importance of the underlying REM sleep regulatory substrate to HVS generation and duration and to the cortico-hippocampal synchronizations of EEG oscillations in hemiparkinsonian rats.

## Introduction

Parkinson’s disease (PD) is a multisystem neurodegenerative syndrome with a significant heterogeneity of motor and non-motor features, whose prevalence increases with age (Müller et al., 2013; Blesa and Przedborski, 2014). The main symptoms of PD are manifested in the motor sphere and include tremors, bradykinesia, muscle rigidity and slowness of movements (Andrzejewski et al., 2016), whereas the non-motor manifestations include cognitive changes, depression, memory impairment, dementia, impaired olfaction and sleep disorders (Afifi and Bergman, 2005; Rodriguez-Oroz et al., 2009).

The progressive neurodegeneration of the SNpc dopaminergic neurons is considered to comprise the pathological substrate for the classical motor symptoms of PD (Pienaar and van de Berg, 2013). Although in most reviews of PD, it is stated that the motor signs first appear when about 50% of the substantia nigra (SN) dopaminergic neurons are lost, there is a good consistency in the available data which suggest that the motor signs appear when there is about a 30% loss of the total SN neurons in comparison to age-matched controls, or when about 80% of the striatal or putaminal dopamine (DA) is lost (Cheng et al., 2010). Beside the well-known reductions in dopaminergic pathways, there is also evidence for alterations in the pedunculopontine tegmental nucleus (PPT) cholinergic pathways in PD, which are associated with impaired cognition, frequent falling, gait problems and sleep disorders (Bohnen and Albin, 2011; Bohnen et al., 2012; Müller et al., 2013; Petrovic et al., 2013a,b, 2014; Ciric et al., 2016, 2017, 2018). Furthermore, there is evidence that PD is preceded by REM sleep behavioral disorders (RBD) prior to the dopaminergic neuropathology, and that these REM sleep alterations are associated with the neuronal loss of the REM sleep regulatory neuronal population, such as the sublaterodorsal nucleus (Garcia-Lorenzo et al., 2013; Boucetta et al., 2016).

Animal models are the best tools to study the mechanisms of PD pathogenesis, and the selection of each particular animal model is very important and dependent on the specific goals of the experimental study (Blesa and Przedborski, 2014). One of the most used animal models of PD is the 6-OHDA lesion of the nigrostriatal DA neurons in rats (Ge et al., 2012; Blesa and Przedborski, 2014). For this rat model of PD, 6-OHDA, as a selective catecholaminergic neurotoxin, is injected unilaterally into the SNpc, the medial forebrain bundle, the striatum (Blandini et al., 2008; Blesa and Przedborski, 2014), or intraventricularly (Rodriguez et al., 2001). All these toxin-based animal models are suitable for studying sleep disturbances, neuropsychiatric and cognitive deficits, alongside the non-motor symptoms observed in PD that are increasingly recognized as relevant to the prodromal disease-state (McDowell and Chesselet, 2012; Blesa and Przedborski, 2014).

The rhythmic oscillations of the neuronal populations [hippocampal theta activity, occipital alpha waves, sleep spindles (SSs)] may serve important physiological functions or may underlie the basis of neurological diseases and rhythmic movement disorders (Buzsaki et al., 1990). The American Academy of Sleep Medicine defined SSs as a “train of distinct waves which having a frequency of 11–16 Hz with a duration ≥0.5 s, usually maximal in amplitude over central brain regions” (Iber et al., 2007). Moreover, abnormally synchronized oscillatory activity in the cortical-basal ganglia loop is associated with motor deficit in PD (Quiroga-Varela et al., 2013; Yang et al., 2015), such as an abnormal beta synchronization (14–30 Hz) and high voltage sleep spindle (HVS) (5–13 Hz) (Buzsaki and Silva, 2012; Dejean et al., 2012). In addition there is evidence that the lack of regulation of thalamo-cortical circuits is associated with an altered SS and HVS dynamics during REM sleep (Ciric et al., 2017, 2018). Since the SS and HVS result from the interactions of the thalamic reticular, thalamo-cortical, hippocampal and cortical neurons (Buzsaki et al., 1990; Steriade, 2006; Piantoni et al., 2016), their characteristics may reflect the integrity of these circuits or serve as the biomarkers of their failing regulation in pathological condition (Warby et al., 2014). Therefore, more attention has been paid to SS and HVS dynamics alterations, notably in relation to various diseases such as schizophrenia, epilepsy, autism, neurodegenerative diseases, and REM sleep behavioral disorder (RBD) (Ge et al., 2012; Urakami et al., 2012; Christensen et al., 2014; Sullivan et al., 2014; Yang et al., 2015).

Recent studies have shown that HVS were enhanced in the neostriatum and substantia nigra pars reticulata (Dejean et al., 2007), the globus pallidus (Ge et al., 2012) and subthalamic nucleus (Magill et al., 2005) after dopamine depletion. It has also been shown that SNpc dopamine depletion in 6-OHDA lesioned rats increase the density and duration of HVS, as a special pattern of spindle activity reflecting the state of the thalamo-cortical regulatory network during PD (Yang et al., 2015). In addition, there is also evidence for the role of dopaminergic neurotransmission in regulating the sleep/wake cycle (Dahan et al., 2007) as well as lesions of SNpc reducing the time spent in REM sleep (Lima et al., 2007, 2008). Moreover, NREM sleep disorder in the hippocampus alongside an altered HVS dynamic during REM sleep in the hippocampus and motor cortex (MCx) have recently been demonstrated as the earliest and long-lasting biomarkers of RBD, expressed earlier than hypokinesia, in the rat model of PD cholinopathy (Ciric et al., 2018). In addition, the altered SS dynamic has been documented in coma, epilepsy, stroke, and several forms of mental diseases (Sullivan et al., 2014), while the HVS are associated with memory impairment and the progression of brain dysfunction (Radek et al., 1994; Sales-Carbonell et al., 2013). In the rat model of PD cholinopathy, during REM sleep, the altered SS dynamic in the MCx and HVS dynamic in the MCx and hippocampus reflected the thalamo-cortical dysregulation (Ciric et al., 2017, 2018).

Therefore, on the basis of all the aforementioned studies, particularly our previous studies in the rat model of PD cholinopathy, which demonstrated the sleep-related alteration of EEG oscillations, cortical drives, and SSs, as the earliest functional biomarkers preceding hypokinesia, our aim in this study was to follow the impact of a unilateral SNpc lesion in rats (specifically hemiparkinsonian rats) on the cortical and hippocampal sleep/wake state architectures, the EEG microstructures, the SS/HVS dynamics and the cortico-hippocampal synchronization of EEG oscillations along with the spontaneous locomotor activity in different sleep states, in order to further investigate the possible earliest functional biomarkers of PD.

## Materials and Methods

### Experimental Design

In this study the experiments were performed on 33 adult male Wistar rats, which were 2-and-a-half month old at the beginning of the study. We followed sleep, basal locomotor activity and spatial memory abilities throughout the same aging follow-up period (from 14 to 42 days following the surgical procedure for the implantation of EEG and electromyographic, EMG, electrodes for chronic sleep recording with or without the unilateral SNpc lesion).

All behavioral assessments were performed after a week of the sleep recording session, at each time point, and during the same circadian phase (during inactive circadian phase for rats, from 9 a.m. to 3 p.m.).

We used 27 rats for sleep recording, divided into three groups: the implanted controls (*n* = 10; Control-i), the unilaterally SNpc lesioned rats, using 1 μl of 12 μg/μl of 6-hydroxy dopamine hydrobromide salt (6-OHDA) solution (*n* = 8; 12 μg/1 μl SNpc lesion), and the unilaterally SNpc lesioned rats, using 2 μl of 6 μg/μl of 6-OHDA solution (*n* = 9; 12 μg/2 μl SNpc lesion).

For the basal behavioral assessments, we included an additional control group of untreated animals (*n* = 6; the physiological control – Control-p).

Prior to surgery and throughout the experimental protocol, the animals were maintained on a 12 h light-dark cycle (7 a.m. lights on, 7 p.m. lights off), and were housed at 25°C with free access to food and water.

This study was carried out in accordance with the recommendations of EEC Directive (2010/63/EU) on the protection of animals used for experimental and other scientific purposes, and the protocol was approved by Ethical Committee for the Use of Laboratory Animals of the Institute for Biological Research “Sinisa Stankovic” of the University of Belgrade (Approval No. 2-21/10).

### Surgical Procedure

The surgical procedures employed for the implantation of EEG and EMG electrodes for chronic sleep recording have been described previously (Ciric et al., 2015, 2018) and are outlined below.

We implanted under ketamine/diazepam anesthesia (Zoletil 50, VIRBAC, France, 50 mg/kg; i.p.), in 2-and-a-half month old rats, two epidural stainless-steel screw electrodes for EEG cortical activity from the MCx (MCx; A/P: +1.0 mm from bregma; R/L: 2.0 mm from sagittal suture; D/V: 1.0 mm from the skull), and two stainless-steel teflon-coated wires (Medwire, NY, United States) into the CA1 hippocampal regions (Hipp; A/P: -3.6 mm from bregma; R/L: 2.5 mm from sagittal suture; D/V: 2.5 mm from the brain surface, according to Paxinos and Watson, 2005). “In addition, for assessment of skeletal muscle activity (EMG) we implanted two stainless-steel teflon-coated wires into the dorsal nuchal musculature and a stainless-steel screw electrode in the nasal bone as a ground. All the electrode leads were soldered to a miniature connector plug (39F1401, Newark Electronics, Schaumburg, IL, United States), and the assembly was fixed to the screw electrodes and skull using acrylic dental cement (Biocryl-RN, Galenika a.d. Beograd, Serbia)” (Ciric et al., 2015, 2018).

During the surgical procedure for the implantation of the EEG and EMG electrodes, we performed the unilateral SNpc lesions by using 1 μl of 12 μg/μl or 2 μl of 6 μg/μl 6-OHDA (Sigma-Aldrich, St. Louis, MO, United States), dissolved in ice-cold sterile saline (0.9% NaCl), and supplemented with 0.2% ascorbic acid, which served as an anti-oxidant, into the right SNpc (A/P: -5.3 mm from bregma; R: 2.4 mm from the sagittal suture; D/V: 7.4 mm from the brain surface, according to Paxinos et al., 2009). For the stereotaxically guided microinfusions, we used a Digital Lab Standard Stereotaxic Instrument (Stoelting Co., Europe) with Hamilton syringe (10 μl). The 6-OHDA concentrations/volumes were chosen on the basis of previous studies (Rodriguez et al., 1999; Gilmour et al., 2011; Pienaar and van de Berg, 2013; Oliveira et al., 2017). The microinfusions were introduced using a single pulse of 200 nl/min constant flow rate, over 5 min for 1 μl, or over 10 min for 2 μl microinfusion. To minimize the uptake of 6-OHDA by noradrenergic neurons, 30 min prior to the microinfusion, each rat received a bolus of desipramine hydrochloride (28.42 mg/kg, i.p., Sigma-Aldrich, Germany; pH = 7.4). Following the microinfusion, the Hamilton syringe was left within the local brain tissue for 5 min before its removal from the brain, allowing the 6-OHDA solution to diffuse within the SNpc.

### Recording Procedure

“At the end of surgical procedure, the scalp wounds were sutured and the rats were allowed to recover for 13 days before their adaptation to the recording cable and plexiglass chamber (30 cm × 30 cm × 30 cm) for 1 day” (Petrovic et al., 2013a,b). “The EEG and EMG activities were carried from the connector plug on the rat head by cable, passed through a sealed port on the recording box, and differentially recorded” (Ciric et al., 2015, 2016, 2018). “The differential mode consisted of six inputs (left MCx, right MCx, left Hipp, right Hipp, left EMG, and right EMG), each with a (+) on the left and a (-) on the right side, and all with the same ground (a screw electrode implanted in the nasal bone)”(Ciric et al., 2015, 2017, 2018).

“The EEG and EMG activities were displayed on a computer monitor and stored on disk for further off-line analysis. After conventional amplification and filtering (0.3–100 Hz band pass; A-M System Inc. Model 3600, Carlsborg, WA, United States), the analog data were digitized (at the sampling frequency of 256/s) and recorded for 6 h, during the normal inactive circadian phase for rats (from 9 a.m. to 3 p.m.) using DataWave SciWorks Experimenter Version 8.0 (DataWave Technologies, Longmont, CO, United States)” (Ciric et al., 2015, 2017, 2018). In this study, the sleep recording sessions of spontaneous sleep were done in all rats 14 and 42 days after the surgical procedure and/or the SNpc lesion.

### Behavioral Assessments

Following the week of each sleep recording session, the basal locomotor activity was tested, over three consecutive days, during the same circadian phase as for the previous sleep recording sessions (from 9 a.m. to 3 p.m.).

The behavioral tests (basal locomotor activity, stereotypy-like and vertical activities) were done after 30 min of habituation to the experimental room, over 30 min (Ciric et al., 2018), by open-field test using the Opto-Varimex Auto-Track System (Columbus Instruments, Columbus, OH, United States). For the assessment of basal locomotor activity, we used the distance traveled as a measure. In addition to the basal locomotor activity, monitored for 30 min (intra-session habituation), we also performed the spatial habituation tests (SHT) over three consecutive days, separated by 24 h intervals (inter-session habituation), and at each time point in the follow-up period (14 and 42 days after the surgical procedure of EEG and EMG implantation with or without the unilateral SNpc lesion).

We note here that for all the behavioral assessments we included an additional control group of non-implanted rats for chronic sleep recording and non-lesioned rats (the physiological controls – Control-p), and we used six rats for each experimental group, except for that of the implanted controls (Control-i; the rats only implanted for chronic sleep recording), were it was seven rats.

For the statistical analyses of the basal locomotor activity, we calculated the means of the distance traveled in 5 min periods for the time-line profiles, and the means ± SE for totals for the 30 min periods.

### Tissue Processing and Tyrosine Hydroxylase (TH) Immunohistochemistry for the SNpc Lesion Identification and Quantification

The SNpc dopaminergic neuronal loss was identified by tyrosine hydroxylase (TH) immunohistochemistry. “The rats were deeply anesthetized and perfused transcardially, starting with a vascular rinse until the liver had been cleared (200 ml of 0.9% saline; perfusion speed of 40 ml/min); followed by a 4% paraformaldehyde solution in 0.1 M PBS (200 ml; 100 ml at 40 ml/min, and then 30 ml/min), and finally with the 10% sucrose solution in 0.1 M PBS (200 ml; 30 ml/min). The animals were sacrificed and the brains were extracted, cleared of the meninges and blood vessels, and immersed in 4% paraformaldehyde overnight, and then in 30% sucrose solution for several days. The brains were cut in a coronal plane into 40 μm-thick sections using a cryotome (Leica, Wetzlar, Germany), and the free- floating sections were further stored in a cryoprotective buffer (0.05 M phosphate buffer, 25% glycerol, and 25% ethylene glycol) at -20°C” (Ciric et al., 2017, 2018).

Immunohistochemistry was performed under the same conditions for all the experimental samples, and the degree of dopaminergic neuronal loss was quantified based on the number of TH immunostained cells in the SNpc. “Brain sections were initially thoroughly rinsed with 0.1 M PBS, pH = 7.4. The endogenous peroxidase activity was neutralized using 3% hydrogen peroxide/10% methanol in 0.1 M PBS for 15 min, and the non-specific binding was prevented by 60 min incubation in 5% normal donkey serum (D9663, Sigma-Aldrich, United States)/0.1 M PBS at RT” (Ciric et al., 2018). The sections were further incubated for 48 h at 4°C with a primary mouse monoclonal anti-TH antibody (dil. 1:16000, T2928, Sigma-Aldrich, United States) in a blocking solution with 0.5% Triton X-100, and subsequently for 90 min in donkey anti-mouse IgG-HRP (dil. 1:50, sc-2318, Santa Cruz, United States). Between each immunolabeling step, the sections were washed in fresh 0.1 M PBS (3 × 5 min). Immunoreactive signals were visualized using a diaminobenzidine solution [1% 3,3′-diaminobenzidine (11208, Acros organics)/0.3% hydrogen peroxide/0.1 M PBS]. All the sections were finally mounted on slides, dehydrated in a series of increasing ethanol solutions (Ethanol 70%, 96%, 100%, Zorka Pharma, RS), placed in a clearing agent (Xylene, Zorka Pharma, RS), mounted with DPX (Sigma-Aldrich, United States), coverslipped and examined under a Zeiss Axiovert microscope with a camera. To test immunolabeling specificity, the primary antibodies were omitted in the control experiments.

All the tissue samples of the lesioned brains (three sections for each rat) were grouped into three defined stereotaxic ranges related to the overall SNpc rostro-caudal dimensions. We quantified the dopaminergic neuronal loss by using Image J 1.46 software. Specifically, TH positively stained dopaminergic cells were counted in three 40 μm thick coronal sections for each brain, representing a certain stereotaxic range within the overall rostro-caudal dimension of SNpc. There were three defined stereotaxic ranges: 4.6–5.1; 5.2–5.7; and 5.8–6.3 caudally from bregma. The number of TH positively stained cells was intended to provide an estimate of the unilateral SNpc dopaminergic neuronal loss (the lesioned SNpc/always the right SNpc) vs. its corresponding contralateral control SNpc (always the left SNpc), rather than to determine the absolute numbers of dopaminergic neurons.

Thus, the dopaminergic neuronal loss was expressed for each defined stereotaxic range, for each brain, and across the overall SNpc rostro-caudal dimension, as the percentage difference of TH positively stained cells with respect to its corresponding contralateral control SNpc TH positively stained cells. To be specific, all the percentage differences were expressed with respect to the mean contralateral control absolute numbers for each stereotaxic range of the SNpc, which was taken as 100%.

### Data Analysis

The analysis of the sleep recorded signals was conducted using software developed in MATLAB 6.5 (Petrovic et al., 2013a,b, 2014; Ciric et al., 2015, 2016; Lazic et al., 2015, 2017), and upgraded to MATLAB R2011a.

### Differentiation of the Sleep/Wake States

“We applied Fourier analysis to signals acquired throughout each 6-h recording (2160 10 s Fourier epochs), and each 10 s epoch was differentiated as Wake, NREM or REM state for further analysis of the Wake, NREM and REM related EEG amplitudes of all the conventional frequency bands (δ = 0.3–4 Hz; 𝜃 = 4.1–8 Hz; σ = 10.1–15 Hz; β = 15.1–30 Hz; γ = 30.1–50 Hz) of the control and the SNpc lesioned rats” (Petrovic et al., 2013a,b, 2014).

“First, we extracted all the 10 s Wake epochs from each 6 h recording, based on the product of sigma and theta frequency power on the *y*-axis, and the EMG power on the *x*-axis” (Petrovic et al., 2013a,b, 2014). “Further, the differentiation of NREM and REM 10 s epochs was done using the EMG power on the y- axis, and the delta/theta power ratio on the x-axis” (Petrovic et al., 2013a,b, 2014). “The differentiation of all Wake/NREM/REM epochs was improved by using the logarithmic values of the quantities on both EEG and EMG axes, and was finally achieved using the two clusters *K* means algorithm” (Petrovic et al., 2013a,b, 2014; Ciric et al., 2015, 2016; Lazic et al., 2015, 2017; Saponjic et al., 2016).

In addition, we also analyzed the Wake/NREM/REM episode dynamics, and the average “episode” duration of each state was calculated by concatenated all bouts of each state and by dividing the total duration obtained by the number of all bouts (10, 20, and 30 s). All the values were expressed as means ± SE in minutes. To be specific, we consider as an episode every single 10 s of Wake/NREM/REM (a 10 s “episode”) or a number of the consecutive 10 s epochs of the same state of Wake/NREM/REM (20 or 30 s “episodes,” and so on). Although the classical term “episode” is not entirely appropriate to our form of data analysis (Ciric et al., 2015, 2016; Lazic et al., 2017) we keep this term as it is conventional.

### Sleep State Related EEG Analysis

“To analyze the sleep/wake state related EEG amplitude changes we calculated the group probability density distributions of all the Wake, NREM and REM conventional EEG frequency bands relative amplitudes over 6 h, of each experimental group, using the Probability Density Estimate (PDE) routine supplied with MATLAB R2011a. In order to eliminate any influence from absolute signal amplitude variations on the recordings, we computed the relative Fourier amplitudes” (Petrovic et al., 2013a,b, 2014):

$$\begin{array}{c} {\left(RA\right)}_{b}=\frac{\sum_{b}\mathrm{Amp}}{\sum_{\mathrm{tot}}\mathrm{Amp}},\;b\;=\;\{\delta , \theta , \sigma , \beta , \gamma \}. \end{array}$$

“In this study, we particularly extracted the Wake/NREM/REM 10 s epochs by using the EEG of the MCx and Hipp for sleep architecture analysis as well as for the state-related EEG microstructures analysis from each brain structure by using the PDE” (Petrovic et al., 2013a, 2014; Lazic et al., 2015, 2017; Ciric et al., 2016, 2018; Saponjic et al., 2016).

“In addition, for each sleep/wake state and each frequency band, PDE analysis was performed on the assembles of relative amplitudes (Petrovic et al., 2013a,b; Lazic et al., 2015, 2017) by pooling measured values from all animals belonging to specific experimental group and at each time point of the overall follow-up period (14 and 42 days after the surgical procedure for implantation of EEG and EMG electrodes for chronic sleep recording with or without SNpc lesion). For the statistical analysis of the PDE/6 h of each EEG frequency-specific relative amplitude (RA)_b_ distribution, and in each state, we calculated the relative amplitude means for Wake and REM during each 30 min, while for NREM during each 60 min” (Ciric et al., 2018).

### Sleep State Related Cortico- Hippocampal Coherences Analysis

Furthermore, we analyzed the Wake, NREM and REM cortico-hippocampal coherences at each time point across the entire follow-up period for each experimental group and for all the conventional EEG frequency bands (Ciric et al., 2015, 2016), using the EEGs of MCx and Hipp. The coherence values were calculated using the “cohere” routine of the MATLAB R2011a Signal Processing Toolbox. It actually computes the magnitude squared coherence between the signals *x*(EEG of the MCx) and *y*(EEG of the Hipp) as

$$\begin{array}{c} C_{\mathrm{xy}}(f)=\frac{{\left|P_{\mathrm{xy}}(f)\right|}^{2}}{P_{\mathrm{xx}}(f)P_{\mathrm{yy}}(f)} \end{array}$$

where *P_xy_*(*f*) stands for the cross spectrum of *x* and *y*, while *P_xx_*(*f*) and *P_yy_*(*f*) denote the power spectra of the two signals.

All the *P_xy_, P_xx_*, and *P_yy_* values were determined for every 10 s of each 6 h recording, and for each frequency within the overall 0.3–50 Hz range, with a 0.1 Hz resolution (Ciric et al., 2015, 2016).

Specifically, the previously identified Wake/NREM/REM EEG 10 s epochs were concatenated and pooled for each time point across the follow-up period, for each state, and for each experimental group (Petrovic et al., 2014). Then, the coherence values within each conventional EEG frequency band (δ, 𝜃, σ, β, and γ) were averaged for each spectrum, and finally, their means were calculated, for each state, each time point across the entire follow-up period, and for each experimental group. For the statistical analysis, the mean coherence values were also calculated for every 30 min of Wake and REM, and for every 60 min of NREM (Ciric et al., 2015, 2016).

### Sleep Spindles Analysis

For the analysis of the SSs, we used from each rat 1 h of NREM or REM sleep, extracted always between 3rd and 4th hour of sleep recording. In order to identify the SS or HVS, we combined automatic detection with the visual validation of all the detected SS or HVS for their final visual extraction and analysis. In particular, “the first step for automatic SS and HVS detection during 1 h of NREM or REM sleep for each rat and experimental group was to filter the EEG signals using the 11–17 Hz band pass filter for SS, or 4.1–10 Hz band pass filter for HVS. Then, we applied the Continuous Wavelet Transform with the mother wavelet “cmorl-2” MATLAB R2011a function, providing a complex Morlet wavelet with determined central frequency *f_0_* = 2” (Adamczyk et al., 2015; Ciric et al., 2017, 2018). In addition, all SS had a minimal duration of 0.5 s, while all HVS had a minimal duration of 1 s.

But, “since we automatically detected many false positive or negative SS or HVS, we had to visually correct all automatically detected SS or HVS for their final extraction from each NREM/REM sleep episode of each rat, and each experimental group. Specifically, automatic detection helps us only to mark SS or HVS, but not to extract them accurately in their overall duration (from the onset to the end of oscillation). Therefore, we had to visually correct the detections of SS and HVS, to extract them more accurately for the analysis of their dynamics. We visually scrolled the automatically marked oscillations in the EEG signal from each brain structure (the MCx or hippocampus), and during each previously determined NREM/REM 10 s episode based on the EEG and EMG.

Than we concatenated all visually detected and extracted SS or HVS during NREM or REM of each group of rats for the analysis of SS or HVS dynamic (mean density; mean intrinsic frequency; mean duration per 1 h of NREM and REM sleep)” (Lazic et al., 2018).

### Statistical Analysis

All statistical analyses were performed using a Kruskal–Wallis ANOVA (*X*^2^*-*values) with the Mann–Whitney U (*z*-values) two-tailed *post hoc* test. The accepted level of significance was *p* ≤ 0.05.

## Results

### The Dopaminergic Neuronal Loss of SNpc After Two Different 6-OHDA Microinfusions

**FIGURE 1:**
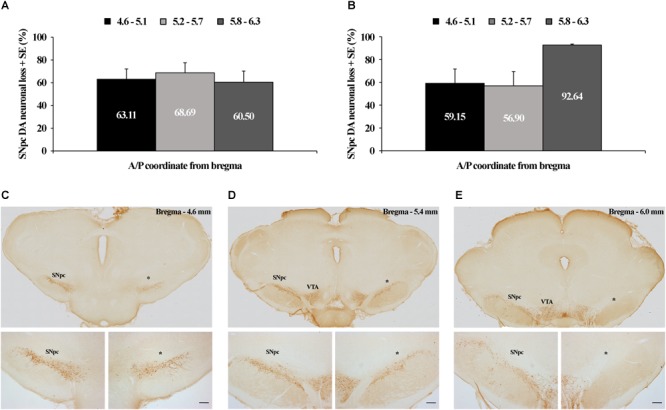
Tyrosine hydroxilase (TH) immunohistochemical identification and quantification of the unilateral substantia nigra pars compacta (SNpc) dopaminergic neuronal loss. Quantified dopaminergic neuronal loss in the unilateral SNpc lesion caused by the microinfusion of 12 μg/1 μl 6-OHDA **(A)** and 12 μg/2 μl 6-OHDA **(B)**. Individual example of three sections used for the quantification of each SNpc lesioned brain **(C–E)**, representing three defined stereotaxic ranges with an identified SNpc partial lesion, caused by 12 μg/1 μl 6-OHDA, throughout the overall rostro-caudal SNpc dimension. The dopaminergic neuronal loss in this SNpc was: 62.30% from 4.6 to 5.1 mm caudally from bregma; 74.47% from 5.2 to 5.7 mm caudally from bregma; and 93.34% from 5.8 to 6.3 mm caudally from bregma. VTA, ventral tegmental area; ^∗^ – indicates the lesioned SNpc; Scale bar 200 μm. The scale bars of the upper **(C–E)** panels, representing only the typical brain sections for the specific SNpc stereotaxic range, are not defined, since they were obtained by the acquisition of the images by using the 10× objective lens of the Olympus BX-51 microscope, equipped with a motorized stage and CCD video camera (Pixelink, Ottawa, ON, Canada), and by using a superimage acquisition option within the newCAST stereological software package (VIS-Visiopharm Integrator System, version 5.3.1.1640; Visiopharm; Denmark).

In this study we have demonstrated that by using two distinct microinfusions, in terms of concentration/volume (12 μg/1 μl of 6-OHDA and 12 μg/2 μl of 6-OHDA), we induced dopaminergic neuronal loss throughout the overall rostro-caudal dimension of the SNpc consistently >60% by using the 12 μg/1 μl microinjection (Figure 1A), while by using the 12 μg/2 μl microinjection it was >56%, from 4.6 to 5.7 mm caudally from bregma, with a maximum loss of >92% most caudally (5.8–6.3 mm caudally from bregma; Figure 1B). The typical individual examples of brain sections used for the quantification of the dopaminergic neuronal loss in SNpc, representing each stereotaxic range within its rostro-caudal dimension, from a rat lesioned by a microinfusion of 12 μg/1 μl of 6-OHDA is depicted in Figure 1C–E.

Since we always quantified the dopaminergic neuronal loss of the lesioned SNpc (the right SNpc) vs. its corresponding contralateral control SNpc (the left SNpc) of each stereotaxic range, those were expressed as percentage differences with respect to the mean contralateral control absolute numbers for each stereotaxic range, taken as 100%; we have to note here that we did not find a statistical difference (*z* ≥-1.89; *p* ≥ 0.07) between the control absolute number of dopaminergic cells at any level of the SNpc rostro-caudal dimension between the two experimental groups (12 μg/1 μl of 6-OHDA and 12 μg/2 μl of 6-OHDA).

### The Impact of SNpc Dopaminergic Neuronal Loss on the Sleep Architecture and Wake/NREM/REM Episode Dynamics

Our results showed that the unilateral lesion of SNpc in both experimental groups of rats (rats lesioned by 12 μg/1 μl of 6-OHDA or by 12 μg/2 μl of 6-OHDA), consistently increased Wake duration, during their normal inactive circadian phase, between 14 and 42 days following the SNpc lesion (*X*^2^ ≥ 6.02; *p* ≤ 0.05; *z* ≥-2.84; *p* ≤ 0.05; Table 1) in the MCx, and likewise in the Hipp. On the other hand, the NREM and REM durations were unaltered from 14 to 42 days following the SNpc lesion (*X*^2^ ≥ 1.92; *p* ≥ 0.18; *z* ≥-1.72; *p* ≥ 0.09; Table 1) in the MCx, and the same was true in the Hipp.

**Table 1:** The impact of SNpc dopaminergic neuronal loss on the Wake/NREM/REM durations and their episode dynamics at 14 and 42 days following the SNpc lesions vs. controls in the motor cortex (MCx) and hippocampus (Hipp): the mean duration of Wake/NREM/REM sleep (minutes) with the mean numbers and mean durations (minutes) of the Wake/NREM/REM episodes per 6 h of sleep ± SE.

				Mean number of			Mean duration of		
	Mean duration/6 h ±*SE* (min)			episodes/6 h ±*SE*			episodes/6 h ±*SE* (min)		
	Wake	NREM	REM	Wake	NREM	REM	Wake	NREM	REM
**MCx 14 days**									
Control	55.24 ± 3.97	205.50 ± 12.88	98.93 ± 10.52	117.14 ± 9.68	310.57 ± 31.04	259.43 ± 33.09	0.48 ± 0.03	0.73 ± 0.12	0.40 ± 0.04
12 μg/1 μl SNpc lesion	**77.69 ± 4.27**	187.93 ± 10.58	94.05 ± 9.70	**152.17 ± 10.12**	320.67 ± 28.54	247.67 ± 32.97	0.52 ± 0.05	0.62 ± 0.08	0.38 ± 0.02
12 μg/2 μl SNpc lesion	**89.45 ± 11.99**	185.86 ± 7.70	84.36 ± 13.71	**154.57 ± 12.35**	261.14 ± 23.48	183.57 ± 30.50	**0.67 ± 0.07**	0.77 ± 0.08	0.39 ± 0.03
**Hipp 14 days**									
Control	52.97 ± 4.83	236.20 ± 10.41	70.50 ± 8.41	97.20 ± 4.99	146.00 ± 2.14	78.40 ± 5.02	0.52 ± 0.02	1.71 ± 0.03	0.77 ± 0.08
12 μg/1 μl SNpc lesion	**77.38 ± 2.80**	213.33 ± 9.42	68.95 ± 8.57	**153.00 ± 11.35**	**229.60 ± 21.75**	**199.60 ± 41.80**	0.52 ± 0.03	**0.95 ± 0.15**	**0.41 ± 0.05**
12 μg/2 μl SNpc lesion	**94.48 ± 4.87**	212.00 ± 6.75	53.19 ± 7.44	**176.67 ± 27.13**	**222.17 ± 27.15**	88.83 ± 17.03	0.58 ± 0.06	**1.05 ± 0.13**	0.60 ± 0.09
**MCx 42 days**									
Control	55.12 ± 5.96	189.21 ± 14.15	115.33 ± 18.03	134.00 ± 5.82	354.00 ± 59.40	296.50 ± 70.04	0.42 ± 0.05	0.64 ± 0.21	0.46 ± 0.11
12 μg/1 μl SNpc lesion	**117.25 ± 19.08**	154.08 ± 16.81	88.33 ± 14.72	**231.75 ± 44.95**	342.00 ± 29.68	257.25 ± 42.19	0.52 ± 0.04	0.47 ± 0.08	0.34 ± 0.01
12 μg/2 μl SNpc lesion	**131.60 ± 16.39**	150.47 ± 15.19	77.60 ± 13.64	**211.20 ± 27.77**	353.40 ± 23.77	271.20 ± 33.26	**0.81 ± 0.14**	0.48 ± 0.08	0.33 ± 0.02
**Hipp 42 days**									
Control	60.33 ± 10.49	221.00 ± 12.55	78.33 ± 13.91	120.40 ± 11.03	217.40 ± 14.57	204.00 ± 36.33	0.40 ± 0.03	1.09 ± 0.13	0.43 ± 0.04
12 μg/1 μl SNpc lesion	**89.42 ± 7.22**	211.22 ± 11.47	59.03 ± 12.50	**189.40 ± 22.78**	256.80 ± 23.12	132.00 ± 27.58	**0.50 ± 0.03**	0.90 ± 0.14	0.38 ± 0.05
12 μg/2 μl SNpc lesion	**114.72 ± 19.54**	193.28 ± 14.21	51.67 ± 9.20	**195.83 ± 24.06**	242.83 ± 20.51	135.17 ± 27.05	**0.57 ± 0.04**	0.84 ± 0.12	0.40 ± 0.03

*Bold numbers indicate the statistically significant mean values vs. their corresponding control values at p ≤ 0.05. All comparisons were made using the Mann–Whitney U test*.

Furthermore, the analysis of Wake/NREM/REM episode dynamics showed that this sleep fragmentation was due to a consistent increase in the mean number of Wake episodes from 14 to 42 days following the SNpc lesion in both the MCx and the Hipp (*X*^2^ ≥ 5.93; *p* ≤ 0.05; *z* ≥-2.74; *p* ≤ 0.05; Table 1). In addition, there was a consistent prolongation of Wake episode mean duration only in the MCx, and mainly following the SNpc lesion using 12 μg/2 μl of 6-OHDA (*X*^2^ ≥ 5.65; *p* ≤ 0.05; *z* ≥-2.31; *p* ≤ 0.04; Table 1).

**FIGURE 2:**
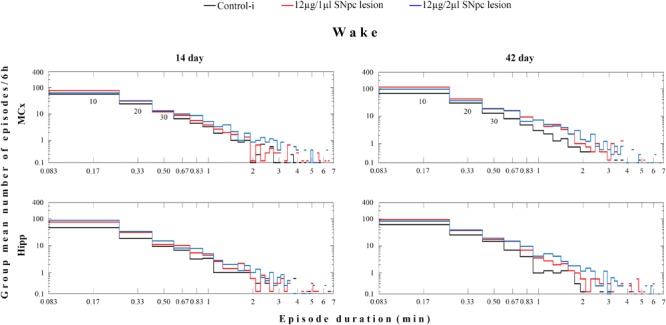
Wake episode dynamics in the MCx and hippocampus (Hipp) at 14 and at 42 days following the SNpc lesion caused by a microinfusion of 12 μg/1 μl 6-OHDA and 12 μg/2 μl 6-OHDA in contrast to the implanted controls (Control-i) - the group distributions of the mean number/6 h of Wake episodes over their durations (minutes). In these log-log distributions each “horizontal stair” is placed above its corresponding duration, depicted on the *x*-axis in minutes – e.g., the group mean number/6 h of 10 s episodes is placed above 10/60 = 0.17 min and so on. The first three episode durations are indicated below the “stair lines” of the two upper panels as 10, 20, 30, presenting the group mean number/6 h of the Wake 10, 20, and 30 s episodes. The missing lines in the distributions depict the zero group mean number of episodes that causes the logarithms to be identified as “not a number.”

However, the group distribution of the mean number of Wake episodes over their durations (in minutes) showed that there was a consistent increase in the number of Wake episodes of all durations in the MCx as well as in the Hipp at each time point during the follow-up period (Figure 2).

### The Impact of SNpc Dopaminergic Neuronal Loss on the Wake/NREM/REM EEG Microstructures

**FIGURE 3:**
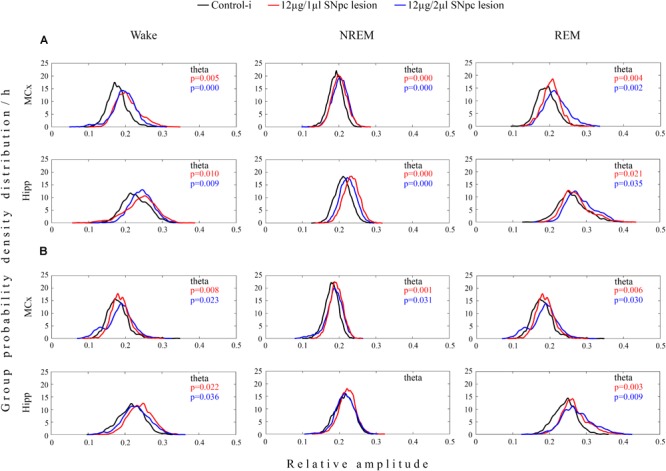
Unilateral dopaminergic neuronal loss in the SNpc augmented theta amplitude across Wake, NREM and REM sleep in the MCx and hippocampus (Hipp) 14 **(A)** and 42 **(B)** days following the SNpc lesion caused by a microinfusion of 12 μg/1 μl 6-OHDA (12 μg/1 μl SNpc lesion) and 12 μg/2 μl 6-OHDA (12 μg/2 μl SNpc lesion). Theta amplitude augmentation following the unilateral SNpc lesion was particularly long-lasting in the MCx and Hipp during both Wake and REM sleep.

In both experimental groups of rats (12 μg/1 μl SNpc lesion or 12 μg/2 μl SNpc lesion) the most consistent EEG microstructure alteration from 14 to 42 days following the SNpc lesion, and across all sleep states, was augmented theta amplitude (Figure 3A,B) in the MCx (*X*^2^ ≥ 5.88; *p* ≤ 0.05; *z* ≥-4.87; *p* ≤ 0.04), and likewise in the Hipp (*X*^2^ ≥ 4.68; *p* ≤ 0.04; *z* ≥-5.12; *p* ≤ 0.04), but which was particularly long-lasting in both structures during both Wake and REM sleep (Figure 3B).

In contrast to the MCx where an augmented theta amplitude, at the onset of the SNpc lesion in both experimental groups of rats, was transiently followed by an attenuated delta amplitude across all sleep states (*X*^2^ ≥ 8.43; *p* ≤ 0.02; *z* ≥-3.22; *p* ≤ 0.02; data not shown), the delta amplitude was not changed in the Hipp (*X*^2^ ≥ 0.73; *p* ≥ 0.11; data not shown).

On the other hand, in the Hipp an augmented theta amplitude was consistently followed by an attenuated sigma amplitude, but only during REM sleep, and only in rats that had been lesioned by 12 μg/2 μl of 6-OHDA (*X*^2^ ≥ 6.36; *p* ≤ 0.04; *z* ≥ -2.29; *p* ≤ 0.04; data not shown).

### The Correlation of the SNpc Dopaminergic Neuronal Loss With the Wake Dynamic Changes and Wake/NREM/REM Theta Amplitudes

When we correlated the means of the Wake duration/Wake episode number of the MCx and Hipp with the dopaminergic neuronal loss, for each defined stereotaxic range of the overall SNpc rostro-caudal dimension, and for each rat of each experimental group (12 μg/1 μl SNpc lesioned rats or 12 μg/2 μl SNpc lesioned rats) we did not find any significant correlation, at any time point over the follow-up period (*r* ≥-0.50; *p* ≥ 0.07). There was only a temporarily significant positive correlation in the Wake prolongation in the Hipp with the SNpc dopaminergic neuronal loss in the 12 μg/1 μl SNpc lesioned rats, 14 days after the lesion (*r* = 0.51; *p* = 0.05; data not shown).

**FIGURE 4:**
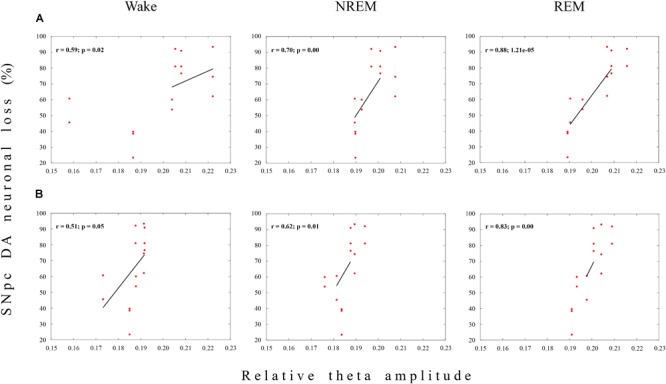
Functional coupling between the dopaminergic neuronal loss of the substantia nigra pars compacta (SNpc) and theta amplitude during Wake, NREM and REM in the MCx. The **s**ignificant positive linear correlations (*r* ≥ 0.51; *p* ≤ 0.05) between the Wake/NREM/REM relative theta amplitudes in the MCx and the dopaminergic (DA) neuronal loss throughout the overall SNpc rostro-caudal dimension at 14 **(A)** and 42 **(B)** days following the SNpc lesion caused by 12 μg/1 μl 6-OHDA (12 μg/1 μl SNpc lesion).

On the other hand, there was a sustained significant positive correlation between the SNpc dopaminergic neuronal loss and theta amplitude across the Wake/NREM/REM of the MCx only in the 12 μg/1 μl SNpc lesioned rats (*r* ≥ 0.51; *p* ≤ 0.05; Figure 4A,B), followed by a significant positive correlation in the Hipp at the onset of the SNpc lesion (14 days after the SNpc lesion), but only during Wake (*r* = 0.81; *p* = 10^-4^, data not shown).

Conversely, this sustained functional coupling between the SNpc dopaminergic neuronal loss and theta amplitude in the MCx, and across Wake, NREM and REM, in the 12 μg/1 μl SNpc lesioned rats (Figure 4A,B) was not present during any sleep state or at any time point of the follow-up period, when we correlated the theta amplitude of the MCx and Hipp with the dopaminergic neuronal loss in the 12 μg/2 μl SNpc lesioned rats (*r* ≥-0.22; *p* ≥ 0.22). In this experimental group, we demonstrated the higher percentage of dopaminergic neuronal loss at the most caudal stereotaxic range of the SNpc rostro-caudal dimension (Figure 1B).

It appears that the dopaminergic neuronal loss of >90% at the most caudal stereotaxic range of the SNpc (about 30% higher than in the 12 μg/1 μl SNpc lesioned rats) uncouples (abolishes) the correlation of the dopaminergic neuronal loss with theta amplitude in the 12 μg/2 μl SNpc lesioned rats, suggesting the importance of the SNpc caudal dopaminergic neurons in theta amplitude control.

### The Impact of SNpc Dopaminergic Neuronal Loss on SS and HVS Dynamics During NREM and REM Sleep

**FIGURE 5:**
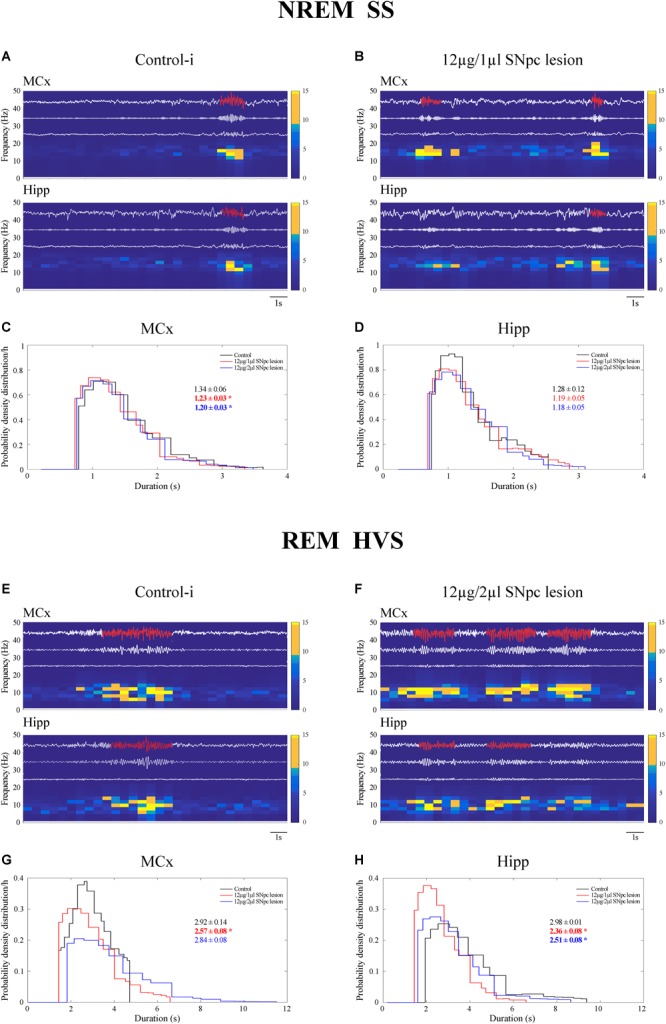
Impact of the unilateral SNpc lesion on the sleep spindles (SS) and high voltage sleep spindles (HVS) dynamics during NREM and REM sleep. Individual examples of SS and HVS during simultaneous NREM and REM sleep in the MCx and hippocampus (Hipp) in the control **(A,E)**, and the SNpc lesioned rats **(B,F)** with their characteristic band pass filters used for their detection and extraction from the EEG signal (11–17 Hz band pass filter for SS; 4.1–10 Hz band pass filter for HVS), the corresponding EMGs and spectrograms, and the impact of the unilateral SNpc lesion on SS **(C,D)** and HVS **(G,H)** durations.

We demonstrate that SS were present mostly during NREM sleep, but that they also occurred during REM sleep in the control rats (Table 2 and Figure 5A). Whereas SS were present in the MCx in ≥80%, they were present in 50% of control rats in the Hipp (Table 2). In the control rats, the SS during REM sleep were longer than during NREM sleep in the MCx and Hipp (Table 2; *z* ≥ -2.25, *p* ≤ 0.05). In addition, the SS during NREM sleep in the Hipp were slower than the SS in the MCx of the control rats (Table 2; *z* = -2.25, *p* = 0.02).

**Table 2:** Sleep spindles (SS) dynamics during NREM and REM sleep.

SS							
MCx	*n*	nSS	Density/h (1/min)	NREM/REM dur/h (min)	SSdur/h (min)	SSdur (s)	SSf (Hz)
**NREM**							
Control	8/10 (80%)	83	0.24 ± 0.03	25.63 ± 2.12	1.80	1.34 ± 0.06	13.25 ± 0.09
12 μg/1 μl SNpc lesion	8/8 (100%)	269	**0.86 ± 0.12**	26.55 ± 1.89	5.50	**1.23 ± 0.03**	13.27 ± 0.05
12 μg/2 μl SNpc lesion	9/9 (100%)	275	**0.70 ± 0.09**	23.23 ± 5.58	5.58	**1.20 ± 0.03**	13.23 ± 0.05
**REM**							
Control	9/10 (90%)	36	0.24 ± 0.03	7.25 ± 0.62	0.96	1.59 ± 0.13**^∗∗^**	13.44 ± 0.16
12 μg/1 μl SNpc lesion	7/8 (88%)	38	0.36 ± 0.06**^∗∗^**	8.69 ± 0.76	0.96	1.52 ± 0.08**^∗∗^**	13.16 ± 0.14
12 μg/2 μl SNpc lesion	8/9 (89%)	29	**0.14 ± 0.02^∗∗^**	8.16 ± 0.85	0.65	1.35 ± 0.09**^∗∗^**	**12.76 ± 0.19^∗∗^**
**Hipp**							
**NREM**							
Control	5/10 (50%)	19	0.05 ± 0.00**^∗^**	25.63 ± 2.12	0.41	1.28 ± 0.12	12.74 ± 0.25**^∗^**
12 μg/1 μl SNpc lesion	8/8 (100%)	121	**0.30 ± 0.04^∗^**	26.55 ± 1.89	2.40	1.19 ± 0.05	12.65 ± 0.07**^∗^**
12 μg/2 μl SNpc lesion	9/9 (100%)	106	**0.29 ± 0.04^∗^**	23.23 ± 5.58	2.08	1.18 ± 0.05	12.71 ± 0.08**^∗^**
**REM**							
Control	5/10 (50%)	10	0.19 ± 0.03**^∗∗^**	7.25 ± 0.62	0.30	1.79 ± 0.21**^∗∗^**	12.90 ± 0.31
12 μg/1 μl SNpc lesion	5/8 (63%)	6	0.11 ± 0.02**^∗^/^∗∗^**	8.69 ± 0.76	0.16	1.56 ± 0.22	12.73 ± 0.28
12 μg/2 μl SNpc lesion	4/9 (44%)	8		8.16 ± 0.85			

*n, number of rats; nSS, total number of extracted SS used for analysis of SS dynamic; density/h, mean frequency of SS per hour of NREM/REM sleep (1/min); NREM/REM dur/h, mean NREM/REM duration (minutes); SSdur/h, total duration of SS extracted during 1 h of NREM/REM sleep (minutes); SS dur, mean SS duration (seconds); SSf, mean SS intrinsic frequency (Hz).*

*Bold numbers indicate the statistically significant mean values vs. their corresponding control values at p ≤ 0.05;****^∗^****indicates statistically significant inter-structure mean values at p ≤ 0.05;****^∗∗^****indicates statistically significant NREM/REM mean values at p ≤ 0.05. All comparisons were made using the Mann–Whitney U test.*

*There were no mean values in the gray rows, and they were not compared, since the SS were present in less than 50% of the rats*.

The unilateral SNpc lesion increased the density of the SS in the MCx, and likewise in the Hipp, only during NREM sleep (Table 2; *z* ≥-3.61, *p* ≤ 0.005), but it decreased their duration (*z* ≥-2.27, *p* ≤ 0.05), with no change in their intrinsic frequency (*z* ≥-1.38, *p* ≥ 0.69), only in the MCx (Table 2 and Figure 5B–D).

In this study, we demonstrate that HVS occurred mainly during the REM sleep of control rats (Table 3 and Figure 5E). As with the SS during NREM sleep in the Hipp (Figure 5A), HVS are slower in the Hipp than in the MCx during the REM sleep of the control rats (Table 3 and Figure 5E; *z* = -2.43, *p* = 0.02).

**Table 3:** High voltage sleep spindles (HVS) dynamic during NREM and REM sleep.

HVS							
				NREM/REM	HVSdur/h		
MCx	n	nHVS	Density/h (1/min)	dur/h (min)	(min)	HVSdur (s)	HVSf (Hz)
**NREM**							
Control	3/10 (30%)	4		25.63 ± 2.12			
12 μg/1 μl SNpc lesion	5/8 (63%)	19	0.08 ± 0.01	26.55 ± 1.89	0.44	1.39 ± 0.07	7.07 ± 0.30
12 μg/2 μl SNpc lesion	3/9 (33%)	11		23.23 ± 5.58			
**REM**							
Control	6/10 (60%)	55	1.01 ± 0.16	7.25 ± 0.62	2.68	2.92 ± 0.14	7.92 ± 0.11
12 μg/1 μl SNpc lesion	8/8 (100%)	230	**1.63 ± 0.16^∗∗^**	8.69 ± 0.76	9.85	**2.57 ± 0.08^∗∗^**	7.92 ± 0.05**^∗∗^**
12 μg/2 μl SNpc lesion	9/9 (100%)	218	**1.43 ± 0.11**	8.16 ± 0.85	9.61	2.84 ± 0.08	7.93 ± 0.05
**Hipp**							
**NREM**							
Control	1/10 (10%)	3		25.63 ± 2.12			
12 μg/1 μl SNpc lesion	1/8 (13%)	1		26.55 ± 1.89			
12 μg/2 μl SNpc lesion	1/9 (11%)	9		23.23 ± 5.58			
**REM**							
Control	6/10 (60%)	60	1.22 ± 0.27	7.25 ± 0.62	2.78	2.98 ± 0.01	7.60 ± 0.09**^∗^**
12 μg/1 μl SNpc lesion	7/8 (88%)	133	0.87 ± 0.10**^∗^**	8.69 ± 0.76	5.23	**2.36 ± 0.08**	7.68 ± 0.08**^∗^**
12 μg/2 μl SNpc lesion	7/9 (78%)	131	1.09 ± 0.18	8.16 ± 0.85	5.05	**2.51 ± 0.08^∗^**	7.67 ± 0.06**^∗^**

*n, number of rats; nHVS, total number of extracted HVS used for analysis of HVS dynamic; density/h, mean frequency of HVS per hour of NREM/REM sleep (1/min); NREM/REM dur/h, mean NREM/REM duration (minutes); HVS dur/h, total duration of HVS extracted during 1 h of NREM/REM sleep (minutes); HVSdur, mean HVS duration (seconds); HVSf, mean HVS intrinsic frequency (Hz).*

*Bold numbers indicate the statistically significant mean values vs. their corresponding control values at p ≤ 0.05; ***^∗^****indicates statistically significant inter-structure mean values at p ≤ 0.05;****^∗∗^*** indicates statistically significant NREM/REM mean values at p ≤ 0.05. All comparisons were made using the Mann–Whitney U test.*

*There were no mean values in the gray rows, and they were not compared since the HVS were present in less than 50% of the rats*.

Moreover, the unilateral SNpc lesion mainly and consistently increased HVS density in the MCx (Table 3 and Figure 5F; *z* ≥-2.29, *p* ≤ 0.03) during REM sleep, alongside their decreased duration, but more consistently in the Hipp (*z* ≥ -4.50, *p* ≤ 0.002), with no change in their intrinsic frequency (Table 3; *z* ≥-1.32, *p* ≥ 0.19).

### The Impact of SNpc Dopaminergic Neuronal Loss on the EEG Oscillations Synchronization Across All Sleep States

**FIGURE 6:**
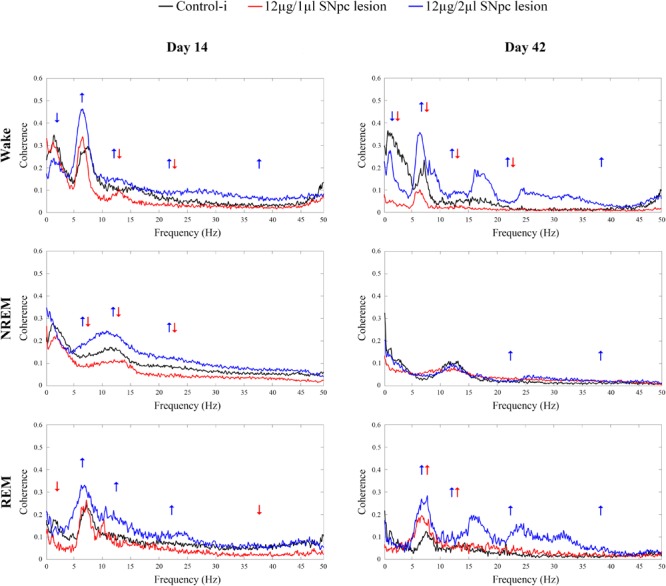
Coherence spectra of all the conventional EEG frequency oscillations between the MCx and hippocampus (Hipp) during Wake, NREM and REM sleep 14 and 42 days following the unilateral SNpc lesions with two distinct microinfusions of 6-OHDA. Arrows indicate the statistically significant mean coherence values at *p* ≤ 0.05, obtained using the Mann–Whitney *U* tests, while their directions indicate the increased or decreased coherences following the unilateral SNpc lesions vs. controls.

We demonstrate that 14 days following the SNpc lesion (Figure 6 Day 14) the theta, sigma and beta synchronizations between the MCx and Hipp increased across all sleep states in the rats lesioned by 12 μg/2 μl of 6-OHDA (*X*^2^ ≥ 5.87; *p* ≤ 0.05; *z* ≥-2.62; *p* ≤ 0.05). Moreover, while delta synchronization decreased, the gamma synchronization increased during Wake (*X*^2^ ≥ 6.42; *p* ≤ 0.04; *z* ≥-3.76; *p* ≤ 0.03).

On the other hand, the sigma and beta synchronizations decreased during Wake and NREM in the rats lesioned with 12 μg/1 μl of 6-OHDA (*X*^2^ ≥ 15.65; *p* ≤ 10^-4^; *z* ≥-2.61; *p* ≤ 0.04). In addition, there was a decreased theta synchronization during NREM (*X*^2^ = 17.05; *p* = 10^-4^; *z* = -2.78; *p* = 0.01), and decreased delta and gamma synchronizations during REM sleep (*X*^2^ ≥ 5.08; *p* ≤ 0.05; *z* ≥-2.31; *p* ≤ 0.05).

During Wake, 42 days after the SNpc lesion using 12 μg/2 μl of 6-OHDA (Figure 6 Day 42, upper panel), the increased synchronizations of theta, sigma, beta and gamma (*X*^2^ ≥ 16.61; *p* = 10^-4^; *z* ≥-3.61; *p* ≤ 0.04), alongside the decreased delta synchronization (*X*^2^ = 20.45; *p* = 10^-4^; *z* = -2.51; *p* = 0.01) were sustained. Conversely, during Wake, after the SNpc lesion using 12 μg/1 μl of 6-OHDA (Figure 6 Day 42, upper panel), the decreased sigma and beta synchronizations were sustained (*X*^2^ ≥ 16.61; *p* = 10^-4^; z ≥-3.32; *p* ≤ 0.003), alongside the decreased delta and theta synchronizations (*X*^2^ ≥ 20.45; *p* = 10^-4^; *z* ≥-3.64; *p* ≤ 0.002).

However, the most striking result was that the divergences of the theta and sigma coherences (desynchronizations) between the MCx and the Hipp, in the rats lesioned using 12 μg/1 μl vs. 12 μg/2 μl of 6-OHDA, became convergent (synchronized) 42 days after the SNpc lesion, but only during REM sleep (Figure 6 Day 42, bottom panel).

To be specific, even in the cases without change vs. the controls during Wake and REM sleep in the rats lesioned using 12 μg/1 μl of 6-OHDA, the increased theta and sigma synchronizations between MCx and Hipp (*X*^2^ ≥ 8.87; *p* ≤ 0.01; *z* ≥-2.56; *p* ≤ 0.02) became a common feature only during REM sleep, in both lesioned group of rats (Figure 6 Day 42, bottom panel). Besides this phenomenon during REM sleep, there were also increased beta and gamma synchronizations during REM (*X*^2^ ≥ 9.75; *p* ≤ 0.01; *z* ≥-2.66; *p* ≤ 0.01), as well as during NREM (*X*^2^ ≥ 8.09; *p* ≤ 0.02; *z* ≥-3.28; *p* ≤ 0.01), but only in those rats lesioned using 12 μg/2 μl of 6-OHDA.

### The Impact of SNpc Dopaminergic Neuronal Loss on Spontaneous Locomotor Activity

Our results show that the unilateral SNpc lesion did not alter spontaneous basal locomotor activity during the overall follow-up period (from 14 to 42 days after lesion) in either experimental group of SNpc lesioned rats (*X*^2^ ≥ 1.34; *p* ≥ 0.47).

**FIGURE 7:**
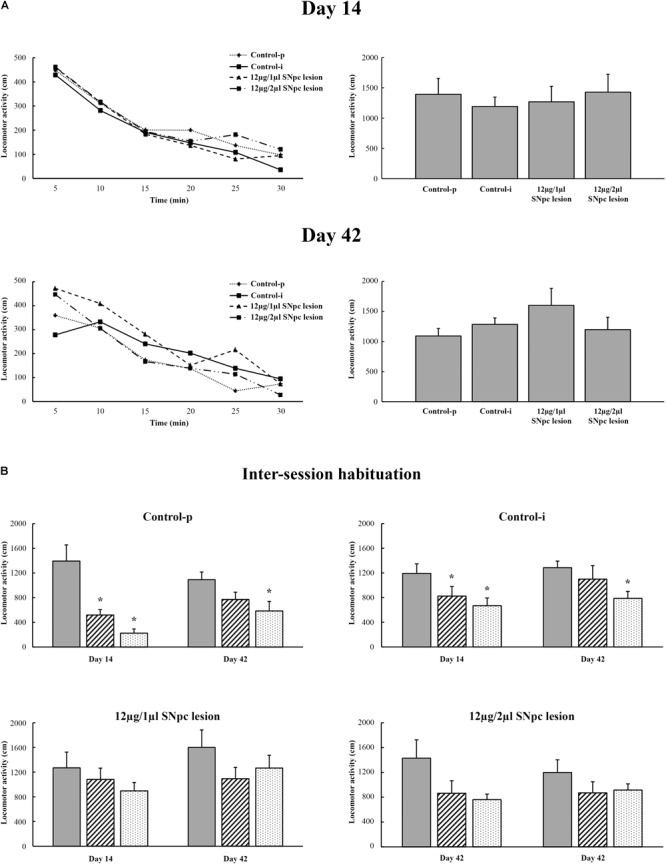
Spontaneous locomotor activity following the unilateral SNpc lesion **(A)** and during inter-session habituation over three consequtive days at each time point **(B)** with respect to the physiological controls (Control-p) and implanted controls (Control-i), expressed as the means for 5 min intervals (left panels) or as the means ± SE for 30 min intervals (right panels) at 14 and at 42 days following the SNpc lesion. The time-dependent profiles of locomotor activity (left panels) were similar for all the experimental groups as well as for the entire registration period (right panels). There was no alteration in the basal locomotor activity. Importantly, in contrast to the physiological controls (Control-p; *n* = 6) and the implanted controls (Control-i; *n* = 7), the time-dependent profiles of locomotor activity during inter-session habituation in the unilaterally SNpc lesioned rats (12 μg/1 μl SNpc lesion, *n* = 6; and 12 μg/2 μl SNpc lesion, *n* = 6), and over three consecutive days, only indicated impaired spatial memory abilities from the onset of SNpc dopaminergic neuronal loss. Stars indicate the statistically significant mean values at *p* ≤ 0.05 obtained using the Mann–Whitney *U* tests.

Figure 7A depicts the means of basal locomotor activity during all the examined time points calculated per 5 min (left panels) and per 30 min intervals (right panels) in all rats. In addition, we did not show any augmentation of stereotypic activity or an alteration in the vertical activity during the overall follow-up period after the SNpc lesion in either group of SNpc lesioned rats (*X*^2^ ≥ 1.23; *p* ≥ 0.24; data not shown).

Although in this study we did not examine memory abilities specifically, we demonstrated the long-lasted alteration of habituatal responses in both groups of SNpc lesioned rats vs. Control-p and vs. Control-i, that indicate impaired spatial memory abilities in the SNpc lesioned rats. Figure 7B depicts the inter-session habitual responses in the Control-p, the Control-i, and in both groups of SNpc lesioned rats. The disordered inter-session habitual responses were present on both Days 14 and 42 in both groups of SNpc lesioned rats (Figure 7B, bottom panels).

## Discussion

Our results show that the unilateral dopaminergic neuronal loss of >50% throughout the overall SNpc rostro-caudal dimension (Figure 1) increased Wake duration, with no change in NREM and REM duration, due to the increased number and duration of Wake episodes in the MCx, as well as in the hippocampus (Table 1 and Figure 2). In addition, there was an augmented theta amplitude across different sleep states in the MCx and in the hippocampus (Figure 3). Moreover, this theta amplitude augmentation, caused by impaired SNpc dopaminergic innervation, lasted throughout the overall follow-up period across Wake/NREM/REM in the MCx (Figure 3A,B), but only during Wake and REM sleep in the hippocampus (Figure 3B, bottom panels). In addition, beside the long-lasting theta amplitude augmentation, there was a sustained positive functional coupling between the theta amplitude augmentation in the MCx and the SNpc dopaminergic neuronal loss across Wake/NREM/REM, but only in the 12 μg/1 μl SNpc lesioned rats (Figure 4A,B).

We have to mention here, that since we recorded sleep for 6 h during the normal inactive circadian phase for rats (from 9 a.m. to 3 p. m.), an increased Wake duration in the hemiparkinsonian rats represents severe sleep fragmentation, not prolonged wakefulness. In addition, we have shown by the analysis of the Wake/NREM/REM episode dynamics that this sleep fragmentation was due to a consistent increase in the mean number of Wake episodes from 14 to 42 days following the SNpc lesion in both the MCx and the Hipp (Table 1). In addition, there was the consistent prolongation of the mean duration of Wake episodes only in the MCx, mainly following the SNpc lesion using 12 μg/2 μl of 6-OHDA (Table 1). However, the group distribution of the mean number of Wake episodes over their durations enable us to show that there was a consistent increase in the Wake episodes number of all durations in the MCx, as well as in the Hipp, for each time point of the follow-up period (Figure 2).

In addition, in order to lesion the SNpc selectively, within the limits of the SNpc, we used two different microinfusions of 6-OHDA in terms of volume (1 μl vs. 2 μl) and concentration (12 μg/μl vs. 6 μg/μl). We induced a significantly higher percentage of dopaminergic neuronal loss only at the most caudal stereotaxic range of the SNpc (92.64%) by using the microinfusion of 12 μg/2 μl 6-OHDA vs. the microinfusion of 12 μg/1 μl 6-OHDA (Figure 1A,B). However, this local, topographically higher dopaminergic neuronal loss did not induce any different effect in the sleep architecture, the sleep/wake state related EEG microstructures, or on the SS or HVS dynamics.

On the other hand, by contrast to the 12 μg/1 μl SNpc lesioned rats, a dopaminergic neuronal loss >90% at the most caudal stereotaxic range of the SNpc rostro-caudal dimension uncoupled (abolished) the correlation of dopaminergic neuronal loss with theta amplitude in the 12 μg/2 μl SNpc lesioned rats, suggesting the importance of the caudal dopaminergic neurons of SNpc in theta amplitude control.

This casually higher percentage of dopaminergic neuronal loss is probably a consequence of the doubled volume of microinfusion alongside the still possible different stereotaxic positioning of the microinfusion needle in the SNpc, due to the individual differences between rats, despite the fact that we used the appropriate and identical stereotaxic coordinates and age of rats in both experimental groups. There is evidence that the volume of microinfusion is more critical than the concentration for any pharmacological stimulation or lesion *in vivo*, or in other words, for a “selective” recruitment of a certain neuronal population within the brain (Bjelobaba and Saponjic, 2013).

In contrast to the rat model of hemiparkinsonism, that we used in this study, our previous studies in the rat model of PD cholinopathy (a severely impaired PPT cholinergic thalamo-cortical innervation; bilateral PPT lesion) demonstrated the topographically distinct EEG microstructures in the sensorimotor and MCx during NREM and REM sleep, expressed mainly as an attenuated delta vs. augmented beta amplitudes during Wake and NREM sleep, and as augmented beta and gamma amplitudes during REM sleep, with no changes in sleep architecture (Petrovic et al., 2013a,b). Beside the augmented cortical activation, the bilateral PPT cholinergic neuronal loss >20% across the overall PPT rostro-caudal dimension, for each brain side, sustainably increased the Wake/REM and REM/Wake transitions followed by increased NREM/REM and REM/NREM transitions (Petrovic et al., 2013a,b), and induced the emergence of two REM sleep states, particularly in the MCx (Petrovic et al., 2013b, 2014). These two REM states were with differential sensorimotor and motor cortical drives to the dorsal nuchal muscles, mainly expressed as impaired beta oscillation drive (Petrovic et al., 2014). In addition, the hallmarks of an earlier aging onset in the rat model of PD cholinopathy was expressed as a unique REM sleep phenomenon – REM “enriched” with SSs of a different pattern, prolonged duration, and slower intrinsic frequency in the MCx as well as the altered motor cortical drive (Ciric et al., 2016, 2017). Moreover, one recent study in the rat model of PD cholinopathy did not show any change in the sleep architecture, but demonstrated a severe and long-lasting EEG microstructure disorder during NREM sleep in the hippocampus, expressed earlier than hypokinesia (Ciric et al., 2018). This long-lasting hippocampal NREM sleep disorder was expressed mainly, from the onset, as delta amplitude augmentation vs. beta amplitude attenuation, and became a common feature of NREM sleep in the hippocampus and MCx, at the end of follow-up period, 91 days after the bilateral PPT lesion (Ciric et al., 2018).

All aforementioned distinct sleep disorders or the differential impact of the impaired SNpc dopaminergic innervation vs. the impaired PPT cholinergic thalamo-cortical innervation on the sleep architectures and sleep state related EEG microstructues demonstrate the important and distinct regulatory roles of both neurotransmitter systems in PD neuropathology. The tonically augmented theta amplitude across all sleep states in the MCx and during Wake and REM in the hippocampus, caused by the SNpc dopaminergic neuronal loss, suggests the importance of dopaminergic innervation for the theta rhythm generation control.

Moreover, in this study we demonstrate that although SS are the hallmarks of NREM sleep, they also occur during REM sleep in the MCx and hippocampus of control rats. In addition, whereas the SS were always longer during REM sleep than during NREM sleep in the MCx and hippocampus, they were consistently slower in the hippocampus (Table 2). The unilateral SNpc dopaminergic neuronal loss increased the density of the SS in the MCx and hippocampus and shortened the SS in the MCx during NREM sleep, but did not change their intrinsic frequency (Table 2 and Figure 5A–D).

In addition, our present results show that HVS are the hallmarks of REM sleep in control rats and that they are, as in the SS, slower in the hippocamus than in the MCx (Table 3). The unilateral SNpc dopaminergic neuronal loss increased the density of HVS in the MCx and consistently shortened HVS in the hippocampus during REM sleep without any alteration in their intrinsic frequency (Table 3 and Figure 5E–H).

Our present study demonstrates that both the SS pattern and duration are state dependent, whereas their intrinsic frequency is brain structure dependent. Beside the fact that the HVS are typical of REM sleep and SS are typical of NREM sleep, both types of SSs were longer when they occurred during REM sleep, indicating the importance of the REM sleep neurochemical regulatory substrate for SS generation and duration control, or for the control of the reticulo-thalamic (RT)–thalamo-cortical (TC)–cortico-thalamic (CT) neuronal loop. In addition, both types of SSs were slower within the hippocampus vs. the MCx, during the state where they typically occurr (NREM or REM), indicating the important regulatory impact of the local neuronal network on the intrinsic frequency of both SSs types.

We should note here that as sleep depth progresses, the RT neurons begin to fire in bursts, generating massive inhibitory postsynaptic potentials in the TC neurons (Piantoni et al., 2016). While the RT neurons plays the central role of pacemaker in the generation of SS oscillation, the reciprocal connections between excitatory and inhibitory neurons in the thalamus and between TC and CT neurons are essential in defining the spatiotemporal dynamics of spindle activity (Piantoni et al., 2016). On the other hand, the duration of the SSs is determined by the duration of RT neuronal activity, and RT neurons firing sharply dropped before the termination of all spindles (Bartho et al., 2014).

Furthermore, studies in both animal models and humans have described SSs as being highly synchronized between multiple cortical regions, but recent human studies have demonstrated that SSs are isolated events (Piantoni et al., 2016). In addition, the phase-locked firing of the hippocampal cells associated with locally recorded SSs in sleeping rat raises the possibility that similar mechanisms are involved in the generation of hippocampal theta and SS activity (Buzsaki et al., 1994). There is also evidence for increased functional connectivity between the hippocampus and the cortex during SSs (Andrade et al., 2011). Furthermore, the hippocampal spindles arise from a circuitry involving the RT and the anterior thalamic nucleus, with which the hippocampus shares reciprocal connections (Aggleton et al., 2010), and they are more spatially restricted and with lower synchrony (Frauscher et al., 2015), suggesting that very local pathways connect the thalamus to the hippocampus. Moreover, it has also been shown that hippocampal SSs are a physiological sleep-related phenomenon and that they precede by several minutes neocortical sleep onset in humans, with increasing delays along the cortical rostro-caudal axis (Sarasso et al., 2014). It has also been demonstrated that during SSs, the cortex is functionally “deafferented” from its hippocampal inputs, due to the bursting mode of activity of the thalamic inputs that recruit massive intracortical inhibitory neurons, which itself produces a local deafferentation of the cortical area concerned, regardless of the input source (Peyrache et al., 2011).

Since in our present study the unilateral SNpc lesion increased the density of the SS during NREM, and likewise of the HVS during REM sleep, but shortened the SS in the MCx and the HVS in both the MCx and the hippocampus, it seems that the dopaminergic neuronal loss potentiates SS generation at the cortical and hippocampal level, but shortened them throughout the frequently potentiated inhibition of the RT neurons. On the other hand, one recent study demonstrated an altered HVS dynamic (prolonged HVS in the MCx with no change of intrinsic frequency vs. shortened and slower HVS in the hippocampus) during REM sleep in the hippocampus and MCx, as the hallmark of PPT thalamo-cortical cholinergic dysregulation in the rat model of PD cholinopathy (Ciric et al., 2018). In accordance with that, our present results show that the impacts of impaired SNpc dopaminergic and PPT thalamo-cortical cholinergic innervations are equally expressed in terms of HVS generation at the motor cortical level (increased density), and in terms of HVS duration at the hippocampal level (decreased duration) during REM sleep. Conversely, the impaired PPT thalamo-cortical cholinergic innervation during REM sleep prolonged the HVS duration in the MCx with no change in their intrinsic frequency, while simultaneously decreasing the duration and intrinsic frequency of hippocampal HVS (Ciric et al., 2018).

Anatomically, the basal ganglia and the PPT share many similarities: they are both heterogeneous structures with similar patterns of inputs and outputs, including cortex, thalamus, amygdala and brainstem, and the PPT has a unique connection reciprocity with the basal ganglia (Mena-Segovia et al., 2004). There is evidence for the similar functions of the basal ganglia and PPT including relevance to locomotion, memory consolidation and sleep regulation (Mena-Segovia et al., 2004). In addition, a new circuitry for NREM and REM sleep regulation in the PD was suggested – a triad composed of PPT, SNpc, and striatum (Targa et al., 2016). Furthermore, the functional connectivity of the PPT and deep cerebellar nuclei (Vitale et al., 2016), suggested the PPT as an interface device between the basal ganglia and the cerebellum in both motor control and cognitive functions (Mori et al., 2016). The PPT is a major source of acetylcholine for the SNpc and an important source of nigrostriatal pathway excitation (Mena-Segovia et al., 2008; Dautan et al., 2014; Targa et al., 2016). Although the PPT and laterodorsal tegmental nucleus (LDT) constitute the only external source of cholinergic innervation of the striatal complex (Dautan et al., 2016), the PPT predominantly targets the dorsolateral striatum, whereas the LDT targets the dorsomedial striatum and the nucleus accumbens (Dautan et al., 2014). On the other hand, both the PPT and the hippocampus receive dopaminergic innervation from the SNpc (Breit et al., 2001; Edelmann and Lessmann, 2018; French and Muthusamy, 2018).

Our present results reveal that the impaired SNpc dopaminergic innervation, like the impaired PPT cholinergic innervation, increased the density of both types of SSs, but decreased their duration, suggested a disorder in the thalamo-cortical regulatory network, that was expressed during both NREM and REM sleep. The altered SS dynamics might be due to the tonically decreased dopaminergic impact through the hippocampus, via the PPT, or indirectly through the basal-ganglia-thalamo-cortical loop.

Moreover, our present study demonstrates that the impaired SNpc dopaminergic innervation severely altered the synchronization of the conventional EEG oscillations between the MCx and the hippocampus during different sleep states (Figure 6). We should note here that we did not find different effects on the sleep architecture, sleep state related EEG microstructures, or on the SS and HVS dynamics, due to the topographically distinct dopaminergic neuronal loss following the SNpc lesion using 12 μg/2 μl vs. 12 μg/1 μl of 6-OHDA (a higher percentage of dopaminergic neuronal loss, mostly caudally, in the SNpc; see Figure 1). However, by using the coherence spectra we were able to identify a distinct synchronization between the hippocampus and MCx during different sleep states, between the two groups of SNpc lesioned rats. All the desynchronizations between the MCx and the hippocampus, in different sleep states, caused by the SNpc dopaminergic neuronal loss, particularly the theta and sigma coherences, in the rats lesioned using 12 μg/2 μl vs. 12 μg/1 μl of 6-OHDA, became synchronized 42 days after the SNpc lesion, but only during REM sleep (Figure 6 Day 42, bottom panel). Thus, the increased theta and sigma synchronizations between the MCx and hippocampus became a common feature in both groups of SNpc lesioned rats only during REM sleep (Figure 6 Day 42, bottom panel), probably due to the increased density of the HVS in the MCx and their simultaneous presence in the hippocampus. These results indicate the most caudal part of SNpc as an important source of hippocampal innervation and the REM sleep state as an important regulatory substrate (during REM there is the highest acetylcholine concentration vs. the lowest noradrenergic and serotonergic concentration) that facilitates synchronization between the MCx and hippocampus in the hemiparkinsonian rat. It is important to mention here that thalamo-cortical system controls vigilance states and gates the perception of sensory stimulation (Steriade, 2006), and that the HVS presents an expression of the brain state that facilitates the detection of weak sensory stimulation (Nicolelis and Fanselow, 2002; Wiest and Nicolelis, 2003).

Moreover, in contrast to delayed hypokinesia developed in the PD cholinopathy (Ciric et al., 2018), in the hemiparkinsonian rats, we did not find any alterations to spontaneous locomotor activity (Figure 7A), stereotypic or vertical activity, except a long-lasted alteration of the habituatal response that only indicated their impaired spatial memory abilities (Figure 7B, bottom panels).

We have demonstrated both long-lasting sleep fragmentation and augmented theta amplitude during the different sleep states of the MCx and Hipp in hemiparkinsonian rats. Moreover, the increased density of SS in the MCx and Hipp during NREM, consistently shorter in the Hipp, and the increased density of HVS in the MCx along with the presence of a consistently shorter HVS in the Hipp during REM sleep, pobably leads to the augmentation of theta and sigma synchronizations between the MCx and Hipp during REM sleep. Our results provide novel evidence for an importance of SNpc dopaminergic innervation in sleep regulation, theta rhythm generation, and the control of SSs dynamics, and highlight the important role of the underlying REM sleep neurochemical regulatory substrate in HVS generation and duration, and in the cortico-hippocampal synchronizations of EEG oscillation control in hemiparkinsonian rats.

## Author Contributions

JC and SK conducted the experiments and analyzed the results. MP conducted the TH immunohistochemistry and interpreted the results. JS designed this study, interpreted the results, and wrote the manuscript.

## Conflict of Interest Statement

The authors declare that the research was conducted in the absence of any commercial or financial relationships that could be construed as a potential conflict of interest.
